# Routing Based Multi-Agent System for Network Reliability in the Smart Microgrid

**DOI:** 10.3390/s20102992

**Published:** 2020-05-25

**Authors:** Niharika Singh, Irraivan Elamvazuthi, Perumal Nallagownden, Gobbi Ramasamy, Ajay Jangra

**Affiliations:** 1 Smart Assistive and Rehabilitative Technology (SMART), Research Group, Universiti Teknologi PETRONAS, Perak 32610, Malaysia; niharika_17005183@utp.edu.my; 2 Department of Electrical and Electronics Engineering, Universiti Teknologi PETRONAS, Perak 32610, Malaysia; perumal@utp.edu.my; 3 Faculty of Engineering, Multimedia University, Persiaran Multimedia, Cyberjaya 63100, Selangor, Malaysia; gobbi@mmu.edu.my; 4 University Institute of Engineering and Technology, Kurukshetra University, Thanesar, Haryana 136119, India; ajangra2015@kuk.ac.in

**Keywords:** distributed energy resources (DERs), microgrid, multi-agent system (MAS), network performance, renewable energy sources (RES), smart grid

## Abstract

Microgrids help to achieve power balance and energy allocation optimality for the defined load networks. One of the major challenges associated with microgrids is the design and implementation of a suitable communication-control architecture that can coordinate actions with system operating conditions. In this paper, the focus is to enhance the intelligence of microgrid networks using a multi-agent system while validation is carried out using network performance metrics i.e., delay, throughput, jitter, and queuing. Network performance is analyzed for the small, medium and large scale microgrid using Institute of Electrical and Electronics Engineers (IEEE) test systems. In this paper, multi-agent-based Bellman routing (MABR) is proposed where the Bellman–Ford algorithm serves the system operating conditions to command the actions of multiple agents installed over the overlay microgrid network. The proposed agent-based routing focuses on calculating the shortest path to a given destination to improve network quality and communication reliability. The algorithm is defined for the distributed nature of the microgrid for an ideal communication network and for two cases of fault injected to the network. From this model, up to 35%–43.3% improvement was achieved in the network delay performance based on the Constant Bit Rate (CBR) traffic model for microgrids.

## 1. Introduction

**Figure 1:**
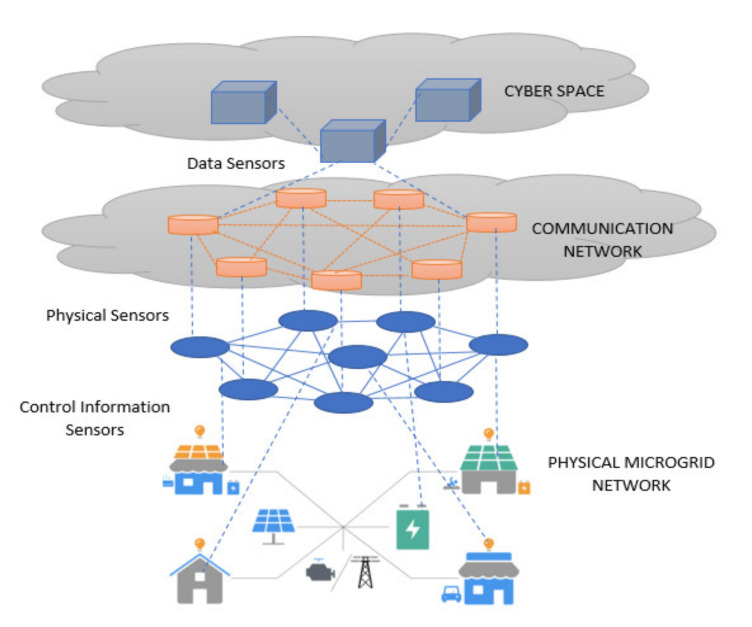
Structure of a microgrid network.

The microgrid is a reliable solution of power systems that offers a plug-and-play interface to harness the potential of integrating numerous renewable generation resources and loads. One of the major challenges associated with microgrids is designing and implementing a suitable communication-control architecture that can coordinate their actions with several system operating conditions. In a microgrid, each node shares information of energy harvesting to the neighboring nodes through energy cooperation [1,2]. In energy harvesting based communications, messages are transmitted through users as per the energy harvested from nature. These systems need carefully designed transmission policies of the users according to the energy arrival profiles [3,4]. Thus, the information flow of the communication network requires a microgrid framework that can fulfill the communication, control, protection and operation requirement without compromising integrity and power network functionality [5,6]. Decision-making in the microgrid considers specific configurations of the communication methods used i.e., cost, degree of availability, data traffic, and number of distributed energy resources (DERs) in the network. Communication configurations in the microgrid are divided into three categories: tightly coupled, loosely coupled, and unicast/broadcast/multicast communications [4,7]. Here, the distributed resources coupled as per the first category (tightly-coupled communications) need the highest possible availability for the network but the other two categories (loosely-coupled and broadcast communications) prefer to manage their operations independently. This implies that the microgrid control system performs in fully or semi- autonomous manner in loosely-coupled and broadcast communications [8,9,10]. Hence, exploring the communication network of a microgrid called “overlay” is an interesting and productive way to improve the technical ability of the microgrid [11]. The network layers of a microgrid can be seen in Figure 1.

For the coverage of a geographical area, the microgrid overlay offers three network designs i.e., Local Area Network (LAN), Metropolitan Area Network (MAN), and Wide Area Network (WAN). LAN configuration in the microgrid works for either case, but in the case of MAN and WAN the configuration expands to broadcast/multicast [12,13]. Hence, to ensure communications to all DERs in the network, the design requirement of Quality of Service (QoS) is a must. In an overlay, with each link node in the network, the delay depends on the bandwidth of the link to carry information. It observes a decrement monotonically with a fixed link bandwidth for the data flowing through it [14]. The reasonable approximation of the information flow in the network is analyzed well using routing techniques [9].

The future of the microgrid demands an intelligent overlay communication network for the microgrid that is self-sustaining and self-sufficient with a significantly prolonged lifetime [3]. There are numerous existing approaches attempting to introduce intelligence within a microgrid. The approaches have different paradigms and technical characteristics for intelligent management. Hence, it is difficult to compare and generalize these approaches [15,16]. An advanced and promising way to achieve this goal is the installation of a Multi-Agent System (MAS). MAS consists of multiple intelligent agents interacting to solve problems that in some cases are beyond the capabilities of a single agent or system [17]. MAS helps in prototyping intelligent control mechanisms over the microgrid and reliably simulates the microgrid operation in the presence of a broad range of devices. In context, the use of a simulator reduces significant investments by avoiding the design of new infrastructure [1,18]. Based on this convention, the proposed approach uses MAS actions triggered by the routing in the microgrid network. In this paper, the focus is on improving quality of service for the microgrid energy network considering throughput, delay optimality, queueing etc. In this paper, data routing and network performance assessment are considered under variable data and energy routing topologies. The work is built upon traditional and recent works on the network routing and topology assignments of the grid communication network. The work aims to assess a microgrid communication network in order to provide energy data cooperation in multi-user network structure.

Contribution points:

1. the proposed methodology identifies the shortest routes to transmit and communicate generation to distribution information data in the microgrid network. This improves network quality based on three parameters i.e., delay, throughput and jitter, along with queueing metrics. The proposed model achieved from 35.3% to 43% improvement in the network delay performance based on the CBR traffic model for the microgrid for the ideal network. In the case of the fault model, the delay improvement varies from 41.1% to 44.5% with improved reliability
2. the paper provides a fault model to improve communication reliability in the microgrid network
3. to validate the proposed algorithm, testing was done for two different cases comparing with three variations in the size of the microgrid i.e., small, medium, and large-scale network

The paper is organized in a manner that explains the outline through an introduction followed by related work as Section 2 describing the research performed in the literature. This section is followed by Section 3, i.e., methodology that explains basic building blocks followed by the flow of the proposed work as Section 4. Further, in Section 5 the simulation environment is described and is followed by a subsection for results and analysis.

## 2. Related Work

**Figure 2:**
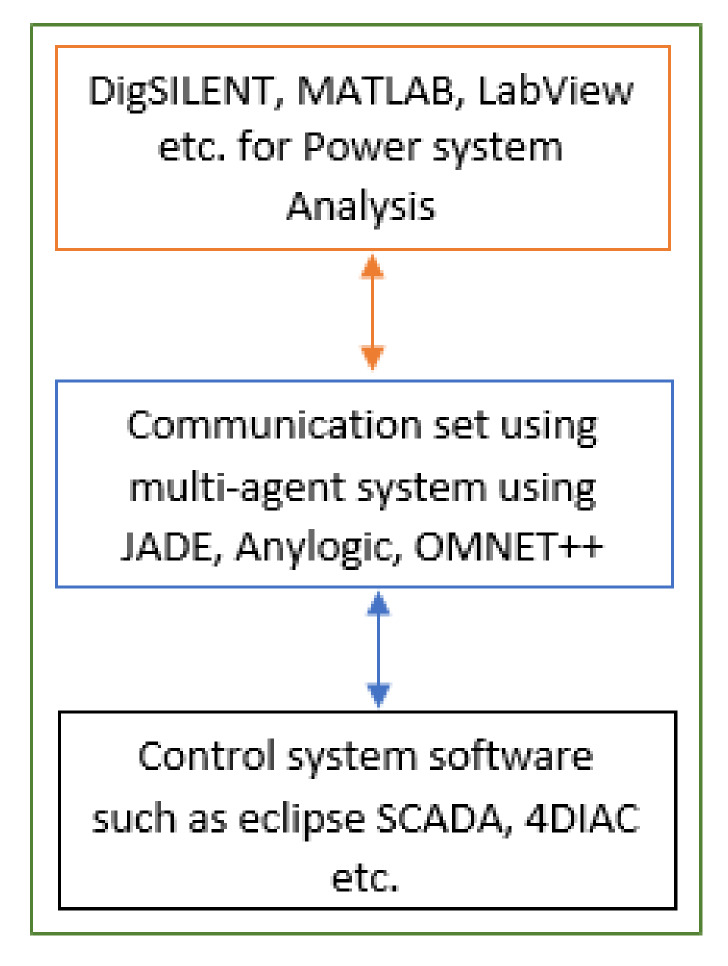
Microgrid communication and control co-simulation approach for communication and control design.

Researchers describe a smart grid as a modernized electricity grid that utilizes information technology and communications to intelligently make automated decisions that help improve resilience, reliability, economics, sustainability, and efficiency of production to the distribution of the grid electricity [19]. In order to make a microgrid smarter and automated several solutions have been published. In ref [19], authors provide an extensive review regarding the impact of Information Communication Technology (ICT) on microgrid performance and enhancing communication control. It also offers prospects of ICT latencies on microgrids. In the field of microgrid communication, artificial Intelligence and machine learning also plays a key role. The techniques are used for schedular designing, resource allocation, integrating communication, and control devices etc. [20,21,22]. In [20], authors have introduced a q-learning based model for resource allocation working over low-latency communication in the microgrid. Another model presented in [21] explains how to reduce the cost of designing a smart microgrid network based on the blocking and intertripping scheme over the physical network. In [23] authors survey the evolving concepts in the microgrid. The need of level-wise communication advancements in the grid network considering primary, secondary, and tertiary levels is explained. It suggests bringing advancements to this industry based on electrical parameters such as networking protocols, standards, communication technologies for interoperability etc. [23,24]. Further, Varna et al. [25], designed a communication architecture for a microgrid that focuses on selection of communication technologies suitable for data transmission in a smart distribution network. The selection of the technology is based upon the several metrics such as delay, packet delivery ratio, bandwidth, cost, throughput etc. The validation of the network is done using an IEEE 5-bus system. Further, the authors concluded that in comparison to an individual or homogeneous approach, a heterogeneous network provides improved performance. Hence, the better approach is to use the ZigBee protocol from the generation hardware unit and wiredLAN is suitable to communicate with the DC-server. Matthias et al. [26], expressed the co-simulation concept between microgrid communication and control. It is a combination of a physical network and a communication network, where the former distributes electricity as per the information given by the latter about demand and supply to/from users/owners. They used three simulators to design the power system model, communication system model, and control system model using DigSILENT, OMNET++, and 4DIAC (IEC 61850) respectively. Figure 2 represents the concept of the microgrid communication and control co-simulation approach used by various researchers for simulation.

4DIAC is built with CLIENT/SERVER service interface using the Transmission Control Protocol/Internet Protocol (TCP/IP) network model. Based on the simulation of the low-voltage microgrid communication network, the behavior of the system evaluated that the impact of bandwidth and the latency of this communication infrastructure are not negligible. Hence, it was concluded that the simulation opens many possibilities to assess the system behavior of communication and information interdependency. Similar co-simulations were performed by Martin et al., [27] using OMNET++ and OpenDSS on the IEEE 13-bus system communicating over 342 households. This research evaluated data-rate-based and event-based sensor communication for electric vehicles. In ref [7], authors say that the role of a microgrid based communication system depends upon the control system design and the component types with their respective counts. They say that the deterministic nature of the microgrid demand and supply, requires information and signal delivery without delays. The research work introduces the integration of several standards and protocols of microgrid communications. The integration of communication systems needs to implement safe, sustainable, reliable, and cost-effective microgrid architecture. Authors suggest two ideas to improve the communication system availability, one is to have system component redundancies and the second is to route data traffic with a combination of a wired and wireless system (this supports the reduction of traffic congestion in wired links by increasing data transfer speeds). The authors concluded that the data delivery delay is higher in information technology networks due to routing and the physical link conversion in the network. The authors also mentioned that there is a scope to find optimal combinations of the network topology and communication protocols in decentralized or agent-based microgrid communication systems.

The operations of a microgrid router (known as energy routers in the case of microgrids) depends upon the information it collects from the grid components. In Ref. [28] authors identify that the microgrid faces two major requirements associated with communication i.e., communication reliability and transmission delay/latency. The communication reliability between these energy routers must be designed with minimized failure probability. Energy routers are required to have a communication failure detection capability in order to quickly retransmit the lost messages [29]. Additionally, when any router encounters equipment failures then other routers in the network should be able to bypass paths to continue communication. On the other hand, these routers must have enough communication capabilities and sufficiently fast processing to guarantee low transmission delay/latency while information exchanges. In a typical case of a critical message these routers expect to have a delivery latency to be as short as 3 ms.

### 2.1. Transmission Latency

A distributed system with MAS enables efficient and effective management of a large or complex distribution network [30]. In ref [31] Duan et al., used a co-simulation architecture based on the Java agent development framework (JADE) to simulate the MAS and the distributed system of a microgrid. The case study model was built using a distribution network in Jiangxi province China. The authors concluded that the communication delay in the network was reduced partially due to the length of the link and the relay agents. They observed that nodes with long queuing delay showed greater response time and communication delay as compared to the nodes with a smaller queue. Han et al. [32], reviewed that the MAS based coordinated controls improves power and energy balance, communication stability, and achieve economic and coordinated operations. They summarized various mathematical and topology models using MAS such as genetic algorithms, non- cooperative game models, graph topology models, and optimization algorithms. They highlighted and summarized communication delay issues in low- or high-bandwidth communication networks. MAS not only serves the wired networks in the microgrids, but the wireless networks as well [33]. Liang et al. noted that MAS based decentralized grid control and communication is a wise choice to avoid a single point of failure. Research presents a multi-agent coordination scheme for the information and data discovery in a microgrid. They concluded that the efficiency of multiagent coordination can be improved using a hybrid/hierarchical network architecture. The authors advised that combined multiagent coordination and multihop routing can be an advanced and futuristic approach to improve microgrid communication. In [34], to provide intelligent energy management and control, Kim and Lim designed a routing-based communication framework using MAS for islanded microgrid with a proactive tree-based routing. The algorithm performs by improving the link quality of the route in the network. The authors expressed the drawback of this work such that the threshold of the link cost affected the network performance and suggested optimizing the performance using a threshold decision scheme in the time-varying environments. In Ref [35], authors designed a locality algorithm based on the peer-to-peer communication infrastructure and aimed to optimize the network performance by improving reliability and latency in the smart microgrid. The authors mentioned that this work could be helpful for future research in communication network optimization of the smart microgrid.

### 2.2. Communication Reliability

Several research ideas have been proposed to improve communication reliability in a microgrid network. In ref. [29], the authors proposed an information and communication network architecture accounting real-time data, availability, scalability, and Quality of service (QoS). The simulation was done using the HIMAP software package. The authors explained that during the 90 s to early 2000 s major network issues occurred due to the failure of the communication and information system, delayed restoration and real-time monitoring as well as the operating control system with a percentage of 32.10%, 38.27%, and 37.04% respectively. The proposed model included a Markov chain, fault trees with component repair, and a Petri net. The authors said that there was a big scope of reliability improvement in their algorithm with an improvement in the redundancy configuration level. In [34], the authors designed a communication framework to operate distributed sources and loads in an islanded microgrid. They employed a mesh network as an advanced topology to reliably deliver the microgrid control frame between agents. The proposed approach reliability was observed with respect to the rate of success on the frame transmission among several appointed agent nodes. The routing approach used to simulate the network path between source and destination is on-demand Ad-hoc On-Demand Distance Vector (AODV). Later, they identified that the routing introduced a higher error rate and reduced link quality. The authors improved the issue and performance up to 6% with better use of the hybrid wireless mesh protocol (HWMP). Further, in [36] the authors devised an event-triggered communication scheme. The testbed used for communication network is 33-bus working in islanded mode.

In [37], Al Suwaidan proposed a communication framework for a self-organized ad-hoc microgrid. The proposed model works on network path building using AODV routing. Further he proposed a fault model and generated two faults in the IEEE 34-bus test distribution network system. The work was based on load routing using the Power line carrier (PLC) network.

With the passage of time, new algorithms are required to improve the microgrid information and communication network. Evaluation of these research works concludes that there is a need to provide a communication model for the microgrid that comprises a hybrid/hierarchical communication (a combination of wired and wireless) using MAS. As per the analysis in the proposed research, a network assessment model was analyzed based on the combination of MAS and a routing algorithm for the hybrid microgrid communication architecture.

Based on related work the proposed model first works to improve network quality based on three parameters i.e., delay, throughput, and jitter, and later provides a fault model to improve communication reliability.

## 3. Network Construction

**Figure 3:**
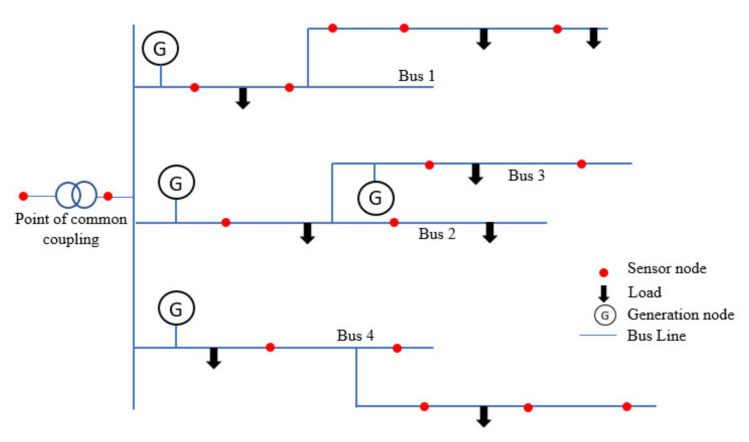
A microgrid physical bus system network.

In a microgrid network, due to high penetration of the distributed generation units, various sensors are deployed over distribution feeders in order to fulfill monitoring and control related tasks. These sensors deliver metered values of phase angles and voltage magnitude, frequency, real and reactive power flow injections, etc. An example of a microgrid network is represented in Figure 3 consisting of several generator nodes, loads, and sensor nodes (highlighted using red color). These sensors are also helpful in acquiring relay and fault statuses in the network by setting links to the communication network as shown in Figure 3.

**Figure 4:**
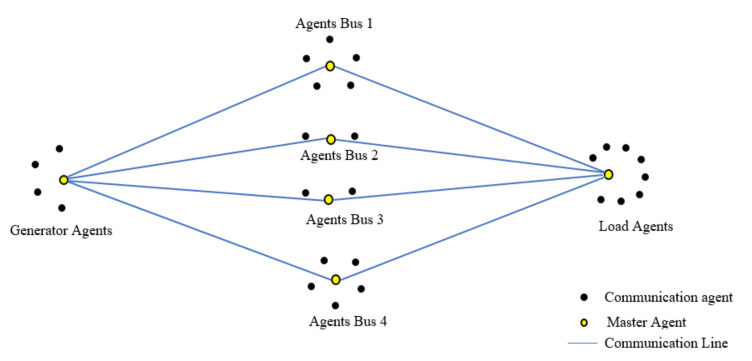
Construction of a microgrid bus system-based communication network.

The microgrid in Figure 3 contains four generators, four bus lines, several sensor nodes, and eight loads. Each bus has a generator source connected to it which supplies electricity to a combination of loads. These buses are connected to a communication network that collects data from the sensors and master agents (highlighted using yellow color) through communication lines. Here in Figure 4, there are four generator agent nodes with a master generator agent, similar to the case for load agents and bus agents. The proposed methodology is a combination of a multiagent system and Bellman–Ford (BF) routing algorithm proposed as multi-agent-based Bellman routing (MABR). Originally, BF was designed and tested to compute the shortest path between a source and destination. Further, it was modified to use for applications with multiple sources and destinations by connecting all sources to a vertex s and destinations to sink t. Based on this motivation, the proposed methodology MABR identifies the shortest routes to transmit and communicate in the microgrid network in order to improve network quality and communication reliability [38].

## 4. Methodology

This section explains the building blocks used during the microgrid network performance assessment. It includes a multi-agent system containing two types of agents i.e., communication agents and physical agents. The subsection is followed by network performance and the algorithm used. Then, it explains various IEEE bus systems, exclusive for the microgrid case study.

### 4.1. Proposed Agent Model

A multi-agent system refers to a team of homogeneous and heterogeneous agents communicating with each other based upon their intelligence (defined algorithm and past experiences) using standard language such as, an agent communication language (ACL), knowledge query and manipulation language (KQML) etc. Generally, agents are categorized based on two types: (i) administrative agents (ii) runtime agents. In the proposed microgrid, agents are classified as physical agents and communication agents. These agents vary based on their attribute nature, i.e., static attributes and dynamic attributes.

The framework considers N physical agents (combination of $\;\sum^{\;}n_{i}$ generator agents deployed to communicate with $\sum^{\;}n_{j}$ load agents) communicating and controlled by M number of communication agents (combination of $m_{\mathrm{master}},{\;m}_{\mathrm{gen}\_\mathrm{host}},{\;m}_{\mathrm{dis}\_\mathrm{tran}\_\mathrm{host}},{\;m}_{\mathrm{load}\_\mathrm{host}}$). Here, $m_{\mathrm{master}}$ is the master communication node for the communication agents $m_{\mathrm{gen}\_\mathrm{host}}$ (host agent for generation controlling agents), ${\;m}_{\mathrm{dis}\_\mathrm{tran}\_\mathrm{host}}$ (host agent for distribution and transmission controlling agents) and $m_{\mathrm{load}\_\mathrm{host}}$(representing host for load agents). Hence, total agents for the framework can be followed as:

(1) Number of agents A=[N+M]

(2) $$A=\left[\left(\;\sum^{\;}n_{i}+\sum^{\;}n_{j}\right)+\left({\;m}_{\mathrm{master}}+m_{{\mathrm{gen}}_{\mathrm{host}}}+{\;m}_{{\mathrm{dis}}_{{\mathrm{tran}}_{\mathrm{host}}}}+{\;m}_{{\mathrm{load}}_{\mathrm{host}}}\right)\right]$$

Based on this equation researchers have analyzed microgrid performance. It communicates based on the heterogenous and homogenous nature of the agents.

### 4.2. Network Performance Assessment

In this paper, the Bellman–Ford algorithm is incorporated in the MAS which helps agents to calculate the shortest path to a given destination. The algorithm is defined for the distributed nature of the microgrid. Bellman–Ford algorithm as follows:

(3) $$D_{i}=\underset{j}{\min}\left[d_{\mathrm{ij}}+D_{j}\right]$$

Here, $D_{i}$ represents the estimation of the shortest distance of the node i to a definite destination. The length between the link (i,j) is designated to $d_{\mathrm{ij}}$. Node i executes the iteration periodically by considering the minimum from neighbor j. $d_{\mathrm{ij}}+D_{j}$ is the estimated shortest distance from node i to the destination passing through j and $\underset{j}{\min}\left[d_{\mathrm{ij}}+D_{j}\right]$ is the shortest distance estimated through the best neighbor.

Several algorithms have been proposed for optimal routing computation of the smart grid but very few for the microgrid data network. In this paper, the authors successfully analyzed the data network for small to large scale microgrids based on the described methodology.

**Figure 5:**
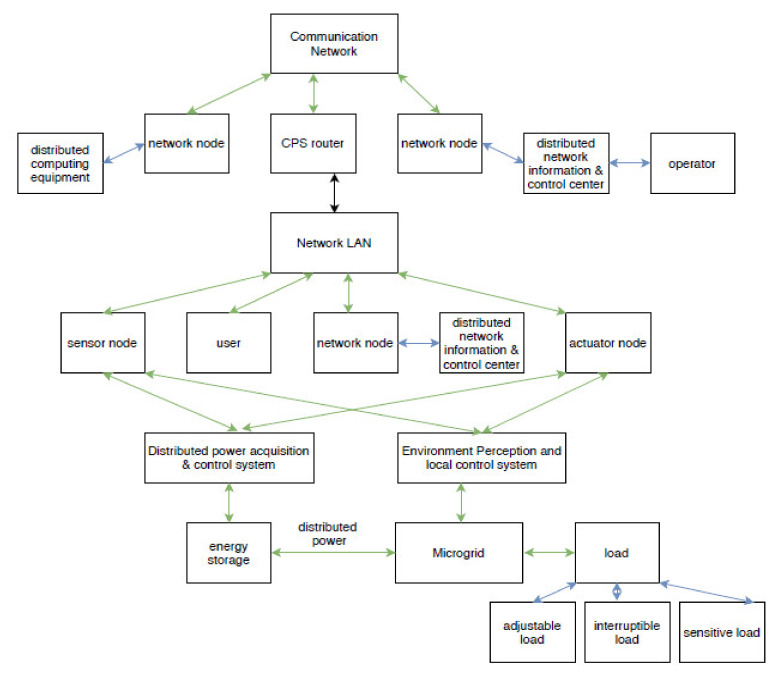
Interactive environment of a microgrid communication network.

In the microgrid, communication and network performance play an important role. The network communication in a microgrid is supported by 100 Mbps ethernet, DNP3 over TCP/IP that may lead to end-to-end delay. Network communication in a microgrid environment can be demonstrated as in Figure 5 [39].

Figure 5 encapsulates the power and communication network of the microgrid. Here, it is not limited to the local microgrid network but also shows how a microgrid interacts with the outside environment. It has network nodes, actuator nodes, and sensor nodes making coordination among distributor, operator, and user [39]. Environment perception and local control system of the microgrid is integrated into the LAN network. It is responsible to handle distributed power and load based on the energy storage and the distributed power acquisition system. To study the behavior of the communication network the Constant Bit Rate (CBR) traffic model [40] is used with uniformly chosen. nodes and traffic flow. The participating nodes are considered as the source and the other nodes as being sunk.

In this microgrid network, routing plays an important role for the network layer protocol to guide the packets from the communication source to their designated destinations. These packets contain information related to the energy flowing in the circuit. Routing in the microgrid involves a complex calculation of algorithms supported by each other, proposing service or information exchange. The complexity of the algorithms is attributed to three causes. First, they require coordination among all microgrid network nodes based on the module as well as their subnet. Second, the microgrid routing needs to cope with node failures and links in order to redirect packets and update the maintained databases. Third, to achieve high performance over congested nodes in the microgrid network. In the microgrid, mainly two performance measures are affected by the routing algorithm i.e., the average packet delay and throughput. The average packet delay refers to the quality of service and throughput refers to the quantity of service. A good routing is responsible for increasing throughput for the same value of average delay per packet during high load demand and a decrement of average delay per packet for low or moderate load conditions.

Network performance can be evaluated based on various parameters, such as packet loss, throughput, bandwidth, delay or latency etc. These measures vary under different stressed conditions. In this paper, the focus is to evaluate the microgrid network based on the end-to-end delay in the network. The protocol and algorithm used for the demonstration is mentioned in the below section.

### 4.3. Proposed Agent Model

Algorithm 1 provides the description of the operating principles of the proposed agent model. Here, it gives several input nodes to the system say X,Y, where X is the physical nodes of the network and Y  is the communication network nodes. This aims to get increment in the network throughput $M_{\mathrm{throughput}},\;$ and reduction in the measure of delay $M_{\mathrm{throughput}},\;$ and jitter $M_{\mathrm{jitter}.}$
**Algorithm 1:** Algorithm for the proposed agent model.**Input:** number of nodes, X,Y **Output:** Increased Throughput measure $M_{\mathrm{throughput}},\;$ and reduced delay $M_{\mathrm{throughput}},\;$ and jitter $M_{\mathrm{jitter}}$
Start; Initialize physical and communication network nodes X and YSet $X=\{x_{1},x_{2},x_{3},\ldots ,x_{p}\}$ and $Y=\{y_{1},y_{2},y_{3},\ldots ,y_{c}\}$For length(X)≥length(Y)Initialize Number of agents A=[N+M]Set $A=\left[\left(\;\sum^{\;}n_{i}+\sum^{\;}n_{j}\right)+\left({\;m}_{\mathrm{master}}+m_{{\mathrm{gen}}_{\mathrm{host}}}+{\;m}_{\mathrm{tran}/{\mathrm{dist}}_{\mathrm{host}}}+{\;m}_{{\mathrm{load}}_{\mathrm{host}}}\right)\right]$Apply agents A over Y communication nodesFor completing task T, agents ${\;m}_{\mathrm{master}}$ divide $T=\{t_{1},t_{2},\ldots t_{i}\}$ and assign A→TWhile $x_{i}$ is waiting for a message length(msg) from $y_{j}$ to make supply and demand decisionApply $D_{i}=\underset{j}{\min}\left[d_{\mathrm{ij}}+D_{j}\right]$,**//**(where $D_{i\;}$is the shortest distance to the node and $d_{\mathrm{ij}}$ is the length between the link (i,j))Agents A finish task T andsend length(msg) to $y_{j}\to x_{i}$End ForCalculate $M_{\mathrm{delay}},{\;M}_{\mathrm{throughput}}$ and $M_{\mathrm{jitter}}$End

The number of physical nodes can be greater than the number of communication nodes. MAS agents are basically situated on the communication nodes arranged in two sets [N+M] where agent set N belongs to the nodes that are sensing data from the various physical sensor nodes. It is a combination of the generation, transmission, distribution, and load sensing units. Further, M is the agent set that has the master node corresponding to those N nodes. The $m_{\mathrm{master}}$ is the master agent that distributes tasks and stores metadata related to the routing topology. Here,${\;m}_{{\mathrm{gen}}_{\mathrm{host}}},$ $m_{\mathrm{tran}/{\mathrm{dist}}_{\mathrm{host}}}$,${\;m}_{{\mathrm{load}}_{\mathrm{host}}}$ are the master host agents that communicate to the generation, transmission/distribution and load agent nodes. Further to perform task T, the $m_{\mathrm{master}}$ divides into several subtasks $T=\{t_{1},t_{2},\ldots t_{i}\}$ and assigns the corresponding agents. This is the point where decisions are made in parallel to the routing play. This task is based upon the length(msg) from the node to the destination node. Further, when the task is completed then the overall performance is measured in terms of $M_{\mathrm{throughput}},{\;M}_{\mathrm{throughput}}$ and $M_{\mathrm{jitter}}$.

### 4.4. Flow Chart

**Figure 6:**
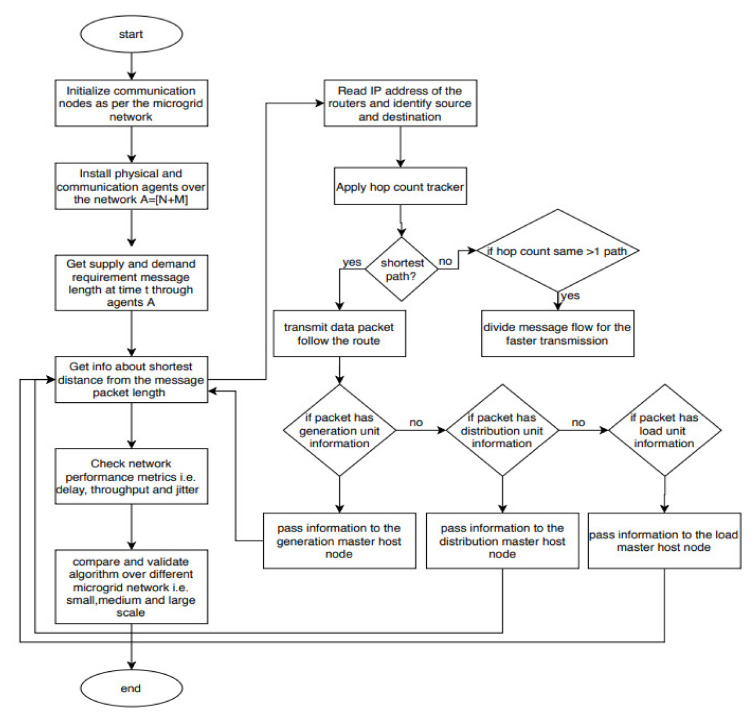
Flow chart for the proposed methodology.

From Figure 6 one can get the complete technical details of the proposed methodology. In Figure 6, initialization of the number of communication nodes is based upon the number of generators, sensors, and loads connected to the microgrid physical network. As per the initialized nodes, an agent model is created as A=[N+M]. Then in this communication network a message of an arbitrary length is shared between agents at time t holding information of demand and supply in the microgrid. Further, these agents identify the shortest distance in the network using the BF algorithm. In order to find the shortest path, IP addresses of the routers are read and identify the source and destination. Then, hop counts are tracked, if it is the shortest path then the packet is transmitted using that route, or if two hop counts are identified with the same value then, the message is divided into segments and transmitted for faster transmission. Further, agents identify that the information packet belongs to which unit, either from generation, distribution, or load and passes to the corresponding master node of the related unit. Based on this communication network, performance metrics are measured i.e., delay, throughput and jitter. These metrics are measured and validated for various microgrid networks considering a scale of small, medium, and large.

## 5. Simulation Results

### 5.1. Performance Metrics

To assess the performance of a microgrid network, several baselines are used. The default baseline is the multi agent system. In addition, the Bellman–Ford algorithm is used as a second baseline which provides routing optimality to the microgrid network. For the MABR simulation network, data/packet streaming uses unicast and broadcast communication channels. The network uses ethernet with 1Gbps network bandwidth (channel capacity). For network performance evaluation, the following metrics are used:

- Throughput: this is the average amount of packets received by the node per second.
  To calculate the throughput at the server:
  Case 1- Session Completed:
  $$\begin{array}{cc} \mathrm{Throughput}\;= & \{(\left[\mathrm{Total}\;\mathrm{Unicast}\;\mathrm{Data}\;\left(\mathrm{Bytes}\right)\;\mathrm{Received}\right]\\ & \;*\;8)/([\mathrm{Last}\;\mathrm{Unicast}\;\mathrm{Message}\;\mathrm{Received}\;–\;\mathrm{First}\;\mathrm{Unicast}\;\mathrm{Message}\;\mathrm{Received}])\} \end{array}$$
  Case2- Session incomplete:
  Throughput ={([Total Unicast Data (Bytes) Received] * 8)/([Simulation Time Parameter–First Unicast Message Received])}
- Delay: this is the function value of travel and processing time of the signal/data packet traversing between sender and receiver:
  E2E−Delay (Average or Mean Delay)={[Total Transmission Delay off All Received Packets]/[Number of Received Packets]}
  where; Transmission Delay of Each Packet =
  {[Time Packet Received at Server–[ Time Packet Transmitted at Client]}
- Jitter: this is the delay inconsistency between each packet. Jitter occurs due to inconsistent delay pacing during packet transmission. To calculate the Average Jitter, the following equation is used:
  Delay−Jitter ={[Total Packet Jitter for all Received Packets]/[ Number of received packet – 1]}
  where;
  Packet Jitter ={[Transmission Delay of Current Packet]–[Transmission Delay o Previous Packet]}

The jitter in the QualNet simulator is defined as Inter Packet Delay Variation (IPDV) (RFC 5481), where the reference is the previous packet in the stream, and the reference changes for each packet in the stream.

IPDV (i)= D (i) –D (i−1); where D (i) denotes the one-way packet delay in the stream.

The ITU standard (Y.1540) has defined Packet Delay Variation (PDV) as: PDV (i)= D (i) – D (min); where D (min) denotes the minimum delay in the stream.

**Figure 7:**
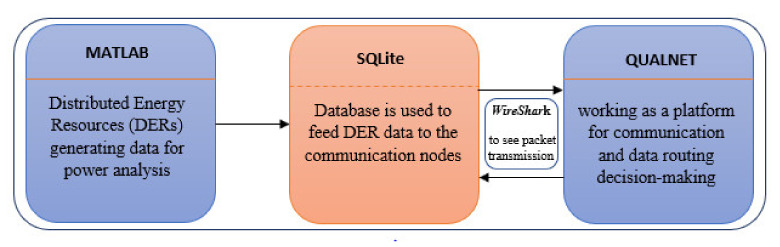
Interconnection of the software modules used for simulation of the grid network.

To simulate the research environment for the proposed algorithm, an interconnection between three software modules was conducted including Matlab, SQLite and Qualnet. Here, Matlab simulates the physical network of the distributed energy resources (DERs) and generates data for decision-making through the communication network. Figure 7 shows the interconnection between simulation software modules.

**Figure 8:**
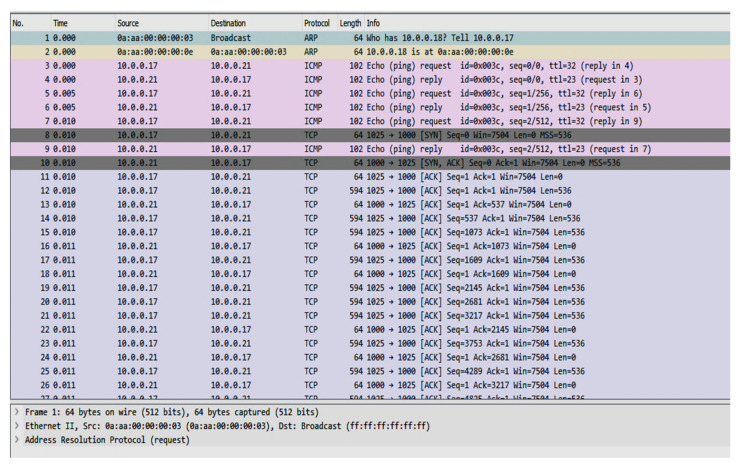
The packet flow for Transmission Control Protocol (TCP) in the microgrid network.

Figure 7 shows that DER data was generated using MATLAB (for example, generation cost from all the active generators and compensators flowing through active buses in the physical network).The data was collected using SQLite to feed the communication nodes installed to setup the communication network in Qualnet for further decision making. Qualnet provides compatibility to the SQLite database which makes it suitable to build an intelligent environment. The communication between SQLite and Qualnet is bidirectional which allows the tester to see the retrieved data/information passing through each node. To see the data packets flowing through the communication network Wireshark was used. It displays messages transmitted in the network along with protocols being used. A snapshot was recorded while communication data was flowing through the network, shown in Figure 8. The sample shot is taken from IEEE 14 communication network.

Figure 8 describes the behavior of various protocols used during the communication process. It explains the involvement of the TCP protocols along with ARP and ICMP. The communication starts with a broadcast packet and receives information through the ARP protocol. For the TCP protocol it uses frame information including the port address, status of SYN or ACK, window size, packet length etc. For communication through the UDP protocol, it uses information about acknowledgement, flag status, length of the packet etc. For the communication model, simulation microgrids are considered as the whole grid with a significantly smaller geographical extent in the literature. Several testing models are provided by the IEEE such as IEEE 4, IEEE 9, IEEE 14, IEEE 33, IEEE 34, IEEE 39, IEEE 72, IEEE 118, IEEE 123 etc. These systems are proposed for the electricity grid testing but have been used for microgrid testing as well [39]. To test the microgrids, electrical lengths are considered to be smaller in comparison to national grid testing. Based on the power production, demands and line length microgrid testing systems are classified into three categories i.e., small, medium, and large. The major difference of using these testing systems is seen for the monitoring, response times, system handling, voltage fluctuations, and less inertia based on the topology. Hence, the scale of the microgrid can be categorized as small microgrids as IEEE 4, 5, 9, 14 etc., medium microgrids as IEEE 23, 33, 34, 39 etc. and large microgrids as IEEE 72, 118, 123 etc.

### 5.2. Result Analysis

**Figure 9:**
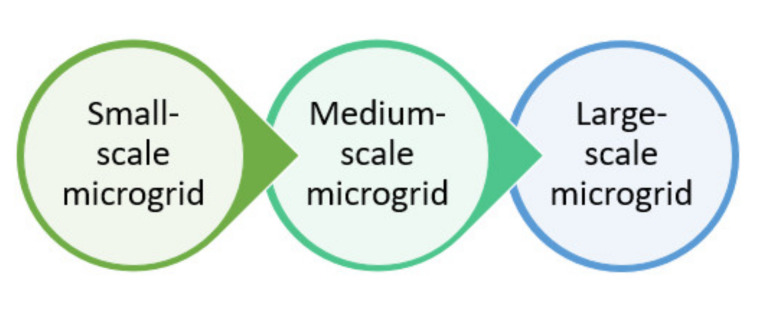
Classification scale of Micro-Grids.

**Figure 10:**
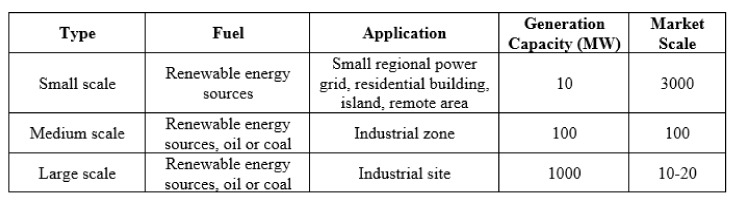
Classifications of micro-grids by Mitsubishi Ltd.

The proposed methodology was simulated over several microgrid networks i.e., IEEE 9, IEEE 14, IEEE 34, IEEE 39. The idea behind simulating over various IEEE systems is the microgrid system classification as shown in Figure 9 and Figure 10. Mitsubishi Ltd. in Japan has classified these systems into small, medium, and large scale [41].

Here, in Figure 10, a small scale microgrid can generate electricity with a capacity of 10 MW using renewable energy resources whereas medium and large scale are capable to producing 100 MW and 1000 MW, respectively [41]. However, medium and large scale obtain fuel not only from renewable energy sources but also from oil or coal. These microgrid scales have different applications such as small scale is capable to feed the small regional power grid, residential buildings, island and remote areas. Further, medium scale and large scale microgrids are capable to feed industrial zones and industrial site applications respectively.

For network simulation, the microgrid uses two ways for data streaming i.e., unicast and multicast upon receiving traffic from the CBR mode. Hence, in Table 1, Table 2, Table 3 and Table 4 three comparisons are made based on the unicast, CBR and broadcast packets. These comparisons are conducted over two approaches i.e., a) algorithms involving no agents and b) the proposed agent-based algorithm. The CBR traffic for IEEE 9 is from node 7 to node 6, for IEEE 14 it is from node 2 to node 14, for IEEE 34 CBR traffic was from node 17 to node 26, for IEEE 39 the traffic is from node 18 to node 15 and for IEEE 57 traffic is from node 38 to node 46. In Table 1, the biggest difference seen is for unicast where no jitter was observed, unlike broadcast data streaming. Performance of the algorithm without agent involvement i.e., Routing Information Protocol (RIP), Optimized Link State Routing (OLSR), Open Shortest Path First (OSPFv2) is slow as compared to the proposed agent-based approach MABR.

In Table 1, for IEEE 9 bus system RIP, OLSR and OSPFv2 shows unicast throughput i.e., 4400, 3050, and 2700 bits/s whereas MABR performed at 3400 bits/s. In this comparison, the RIP protocol serves better throughput. Further, delay performance in unicast is best served by the MABR with 0.00131 s as compared to the 0.0073, 0.0073, and 0.0074 served by the RIP, OLSR, and OSPFv2 respectively. For broadcast performance, OLSR performed better with throughput 2000 bits/s. For delay and jitter, the metrics of the MABR approach gave better results than others with 0.0011 s delay and 5.5 × 10^−8^ s variation jitter between several nodes in the network. Further, other metrics observed are hop count, queue length, and the longest time in the network queue. Here OLSR wins the hop count comparison with a score of 1 but MABR performs best with the minimum queue length of 9 × 10^−6^ bytes and takes the minimum time in the queue i.e., 5 × 10^−6^ as compared to the other algorithms. Hence, the following Table 1 gives performance clarity that the MABR scores best for 6 metrics out of 10 metrics as compared to other algorithms with no agent involvement. Similarly, for the IEEE 14 bus, RIP, OLSR, and OSPFv2 show unicast throughput i.e., 3400, 3050 and 2700 bits/s whereas the proposed MABR performed at 3450. On comparing, MABR serves better throughput. Further, delay performance in unicast is best in MABR approach with 0.0073 s as compared to the 0.0074, 0.0074, and 0.0074 served by the RIP, OLSR, and OSPFv2 respectively. Further, broadcast performance, OLSR performed better with throughput 9700 bits/s. For delay and jitter, the metrics of the MABR approach gave better results than the others with 0.0013 s delay and 0.000019 s variation in jitter. Here OLSR wins the hop count comparison with a score of 1 but the Agent model performed best with the minimum queue length of 0.008 bytes and took the minimum time in the queue i.e., 0.0043 s as compared to the other algorithms. For the IEEE 34 bus system, RIP, OLSR, and OSPFv2 are slow as compared to the proposed agent-based approach. RIP, OLSR, and OSPFv2 show unicast throughput i.e., 2100, 2900, and 1850 bits/s whereas the proposed MABR approach performed at 3450. In this comparison, the proposed MABR approach serves better throughput. Further, delay performance in unicast is best served by the MABR with 0.0145 s as compared to the 0.015, 0.0145, and 0.0155 served by the RIP, OLSR, and OSPFv2 respectively. Also, the difference seen at OLSR showed a jitter of 1.7 × 10^−5^ unlike the other cases. Moving to the broadcast performance, OLSR has performed better with throughput 13,000 bits/s. For delay and jitter, the metrics agent approach gave better results than the others with 0.001 s delay and 0.000185 s variation in jitter between several nodes in the network. Here OLSR wins the hop count comparison with a score of 1 but the Agent model performs best with the minimum queue length of 0.0095 bytes and takes the minimum time in the queue i.e., 0.00055 s as compared to the other algorithms. For the IEEE 39 bus system, RIP, OLSR, OSPFv2 are slow as compared to the MABR approach. RIP, OLSR, and OSPFv2 show unicast throughput i.e., 2900, 3000, and 1850 bits/s whereas the proposed approach performed at 3450. In this comparison, the proposed approach serves better throughput for unicast transmission. Further, delay performance in unicast is best served by the MABR with 0.0013 s as compared to the 0.0073 served by the other cases. Moving to the broadcast performance, OLSR performed better with throughput 46,000 bits/s. For Delay and jitter, the metrics agent approach gave better results than the others with 0.000125 s delay and 0.00015 s variation in jitter between several nodes in the network. Further, other metrics observed are hop count, queue length, and the longest time in the network queue. Here OLSR wins the hop count comparison with a score of 1 but the Agent model performed best with the minimum queue length of 0.01 bytes and took the minimum time in the queue i.e., 0.000457 s as compared to the other algorithms. The simulation response for the large scale microgrid i.e., system IEEE 57 is observed to be similar compared to the small and medium scale microgrid network designs. The delay measured for UDP unicast is 0.00145 s and for broadcast transmission it measured 0.0014 s. Then, response for throughput is better in the case of the proposed multi-agent-based algorithm for unicast and for broadcast the OLSR algorithm performed better. The difference was seen in the case of unicast transmission and CBR traffic as jitter was seen in both cases unlike the broadcast transmission. For other metrics, the proposed algorithm performed better.

The comparative analysis of Table 1 is explained in Table 2 which depicts how MABR is better when compared to other algorithms with no agent involvement RIP, OLSR, and OSPFv2.

Table 2 illustrates that the proposed agent-based approach MABR performed better for 6 metrics out of 10 metrics that are compared. Overall, the proposed approach showed performance improvement by reducing transmission delay up to an average of 43% for unicast delay, 42.3% for CBR traffic model delay, and 35.3% for broadcast delay. The other improvements are seen in the case of unicast throughput with an average of 37.9%. The proposed approach also worked well for the overall message queue length and the results of the longest time metrics were noticed to be minimum for the proposed agent approach as compared to the other algorithms that involve no agent.

Further, network was analyzed by introducing faults in the communication model. Two types of faults were introduced that are explained in the following case studies.

#### 5.2.1. Case Study 1: Network Containing Single Fault

This case study considers a single fault for all the microgrid network systems. The changes are compared with Table 1 and the visible changes are highlighted with red color in Table 3. For IEEE 9 bus system, the fault was injected at node 7, for IEEE 14 the fault was at node 2, for IEEE 34 bus system the fault was injected at node 17, for IEEE 39 the fault was at node 18 and for IEEE 57 the fault was injected at node 38.

For the fault network study, Table 3 shows that the more visible changes (as compared to the ideal case) were at the small and medium network like IEEE 9 and IEEE 14. In the case of IEEE 34 and IEEE 39, the network fault did not affect the network performance much. These effects are based upon the size of the network; if there is only one fault then the proposed algorithm has worked well to find other routes to send information packets to agents. In case of IEEE 9, throughput, delay, hop count, and queueing metrics fluctuated. Further in the case of IEEE 14, throughput, delay, and hop count showed visible changes. For IEEE 34 and IEEE 39 changes were seen at throughput and delay. In the IEEE 57 network, changes were seen in the throughput of OLSR for unicast transmission and some delay reduction in case of RIP Further, the network was analyzed by injecting two faults to each bus system for testing network performance.

#### 5.2.2. Case Study 2: Network Containing Two Faults

Case study 2 considers two faults in each communication network of the microgrid bus systems. The changes are compared with Table 3 and the visible changes are highlighted with violet color in Table 4. For the IEEE 9 bus system, the fault was injected at node 7 and node 8, for IEEE 14 the fault was at node 2 and node 5, for IEEE 34 bus system the fault was injected at node 17 and node 6, also for IEEE 39 the fault was at node 18 and node 17, for the large scale microgrid IEEE 57 the fault was injected at node 38 and node 27. These fault nodes are considered as they block a maximum number of paths when the fault was injected to test all the metrics under critical conditions.

In Table 4, the performance of the algorithms with no agent involvement i.e., RIP, OLSR, OSPFv2 is slower as compared to the proposed agent-based approach even in the fault model. For the IEEE 9 bus system RIP, OLSR, and OSPFv2 show unicast throughput i.e., 4200, 3050, and 2500 bits/s whereas the proposed approach performed at 3500 bits/s. In this comparison, the RIP protocol serves better throughput. Further, delay performance in unicast is best served by the proposed agent-based approach with 0.00131 s as compared to the 0.0074, 0.0073, and 0.0074 served by the RIP, OLSR, and OSPFv2 respectively. The throughput of each algorithm was changed in the fault model as compared to no fault or the ideal case network. In the case of CBR traffic IEEE 9 showed throughput of 4300 bits/s which is less than 4700 bits/s as shown by OSPF, but delay was minimum for the proposed approach i.e., 0.0013 s. Moving to the broadcast performance, OLSR performed better with throughput 2300 bits/s. This throughput measure changed from 2000bit/s to 2300 bits/s as compared to the no fault model for delay and jitter, the metrics agent approach gave better results than others with 0.0011 s delay and 5.5 × 10^−8^ s variation jitter between several nodes in the network. Further, the other metrics observed are hop count, queue length, and the longest time in the network queue. Here OLSR wins the minimum hop count comparison with a score of 2 but the Agent model performs best with the minimum queue length of 9 × 10^−6^ bytes and took the minimum time in the queue i.e., 5 × 10^−6^ as compared to the other algorithms. Similarly, for the IEEE 14 bus, RIP, OLSR, and OSPFv2 show unicast throughput i.e., 3350, 3050, and 2700 bits/s whereas the proposed approach performed at 3450. In this comparison, the proposed approach serves better throughput. Further, the delay performance in unicast is best served by the proposed agent-based approach with 0.0073 s as compared to the 0.0074, 0.0075, and 0.0074 served by the RIP, OLSR, and OSPFv2 respectively. For CBR traffic, the proposed approach showed minimum delay i,e., 0.0072 s. Moving to the broadcast performance, even for this fault model the proposed approach and OLSR performed better with throughput 9000 bits/s. For delay and jitter, the metrics agent approach gave better results than the others with 0.0013 s delay and 0.000019 s variation in jitter between several nodes in the network. Further, other metrics observed are hop count, queue length, and the longest time in the network queue. Here OLSR wins the minimum hop count comparison with a score of 1 but the Agent model performed best with the minimum queue length of 0.008 bytes and took the minimum time in the queue i.e., 0.0043 s as compared to the other algorithms with 0.00044, 0.00062, and 0.0095 s. For the IEEE 34 bus system, RIP, OLSR, OSPFv2 are slower as compared to the proposed agent-based approach. RIP, OLSR, and OSPFv2 show unicast throughput i.e., 2100, 2800, and 1850 bits/s whereas the proposed approach performed at 3450 bits/s. In this comparison, the proposed approach serves better throughput. Further, delay performance in unicast is better served by the proposed agent-based approach and RIP with 0.015 s as compared to the 0.0145 and 0.0155 served by the OLSR and OSPFv2 respectively. Also, the difference seen at OLSR showed a jitter of 1.7 × 10^−5^ unlike other cases. Moving to the broadcast performance, OLSR performed better with throughput 14,000 bits/s. This OLSR throughput in the case of broadcast is even better than the ideal/no fault model. For delay and jitter, the metrics agent approach gave better results than others with 0.001 s delay and 0.000185 s variation in jitter between several nodes in the network. Further, other metrics observed are hop count, queue length, and the longest time in the network queue. Here OLSR wins the hop count comparison with a score of 1. Here, unlike the ideal case that showed a hop count score of 3, this fault model showed a hop count score of 2 for the proposed approach. The proposed agent model performed best with the minimum queue length of 0.0095 bytes and took the minimum time in the queue i.e., 0.00055 s as compared to the other algorithms. For the IEEE 39 bus system, RIP, OLSR, OSPFv2 are slow as compared to the proposed agent-based approach. RIP, OLSR, and OSPFv2 show unicast throughput i.e., 2900, 3100, and 1850 bits/s whereas the proposed approach performed at 3450 which is better than the other approaches. Further, delay performance in unicast is best served by the proposed agent-based approach with 0.0013 s as compared to the 0.00735 and 0.0073 s served by the other cases. Moving to the broadcast performance, OLSR performed better with throughput 45,000 bits/s which is less than the ideal case with 46,000 bits/s. For delay and jitter, the metrics agent approach gave better results than the others with 0.000125 s delay and 0.00015 s variation in jitter between several nodes in the network. Further, other metrics observed are hop count, queue length, and the longest time in the network queue. Here OLSR wins the hop count comparison with minimum score of 1 but the Agent model performs best with the minimum queue length of 0.01 bytes and took the minimum time in the queue i.e., 0.000457 s as compared to the other algorithms. The difference in the ideal case and the fault model for the maximum time in the queue was seen for OSPF with 0.0192 s. For IEEE 57 the changes were seen when the unicast transmission throughput was changed to 3,550 bits/s for the proposed agent algorithm. Along with that, the end to end delay was different i.e., 0.00135 s.

Table 4 shows that the proposed agent-based approach gives a better performance as compared to other approaches. The comparison was made based on the Relative percent difference (RPD). Here, first, the sum of the different measurements was calculated and then divided by the total number of measurements to obtain the average. Then this relative difference was divided by the average of RPD and multiplied by 100 to get the percentage. To understand the concept, for two measurements i.e., $x_{1}$ and $x_{2}$, the overall formula is [($\left|x_{2}-x_{1}\right|$)/{(|$x_{2}+x_{1|}$)/2}], where {(|$x_{2}+x_{1|}$)/2} in the denominator denotes the average of the measurements. Overall for the fault model, the proposed approach showed performance improvement by reducing transmission delay up to an average of 42.6% for unicast delay, 44.5% for CBR traffic model delay, and 41.1% for broadcast delay. The other improvements are seen in the case of unicast throughput with an average of 38.1% and 58.8% in broadcast throughput. Jitter was also improved in the various bus systems on an average of 70.6% in broadcast but could not do better for the unicast and burst traffic model. The proposed approach also worked well for the overall message queue length and the results of longest time metrics were noticed to be the minimum for the proposed agent approach as compared to the other algorithms that involve no agent.

Hence, it can be concluded that the proposed agent-based approach is helpful in improving the network performance and communication reliability of any microgrid network. The proposed agent- based Bellman routing algorithm performed better for transmission latency in the microgrid for small, medium, and large scale as compared to no agent-based algorithms but, in the case of unicast throughput, the RIP protocol worked well for small and medium scale microgrids and in the case of broadcast throughput the OLSR protocol performed well. Hence, in future an algorithm can be designed that may improve all these metric scores. After the use of a multi-agent system researchers can use artificial intelligence or deep-learning in order to improve fault diagnosis and decision-making for the microgrid communication network.

The proposed algorithm makes an appropriate representation to the multi-layered protocol standards such as field equipment IEC 61850, NIST, IEEE 1815 (DNP3) communication etc. To set up the smart microgrid power system, the IEC 61,499 device can be used which comprises several DERs and a networking device grouped together working over protocols. In the real-world scenario, the proposed algorithm would deal with these protocols using its communication services operating with the data based on several attributes, such as the timestamp of the data, quality of the data, and common data classes synced over object-oriented programming etc. This multi-layer interconnection between the microgrid physical network, communication network, proposed algorithm, and several protocols would help in making the grid smarter, named as the Smart Microgrid.

## 6. Conclusions

In this paper, to assess the performance of the microgrid network, several baselines are used. The default baseline is the multi agent system. In addition, the Bellman–Ford algorithm is used as a second baseline which provides routing optimality to the microgrid network. For the microgrid simulation network, data/packet streaming uses the unicast and broadcast communication channels receiving traffic from the CBR mode. The paper compares the proposed agent-based routing approach MABR with various ‘no agent-involvement-based’ routing approaches. In the case of an ideal traffic model, the proposed MABR was able to achieve a performance improvement by reducing the transmission delay up to an average of 43% for the unicast delay, 42.3% for the CBR traffic model delay, and 35.3% for the broadcast delay. The other improvements are seen in the case of unicast throughput with an average of 37.9%. On the other hand, in the case of the fault model, performance improvement was seen with a reduction of transmission delay up to an average of 42.6% for unicast delay, 44.5% for CBR traffic model delay, and 41.1% for broadcast delay. The other improvements are seen in the case of unicast throughput with an average of 38.1% and 58.8% in broadcast throughput. Jitter was also improved in various bus systems on an average of 70.6%. Overall the proposed agent-based approach was able to improve the network performance and communication reliability of the microgrid network.

## Figures and Tables

**Table 1:** Performance evaluation of the bus system for the ideal microgrid communication network.

Microgrid Bus System (100 Mbps Bandwidth)		UDP: Unicast		CBR Server		UDP: Broadcast					
		Throughput (bits/s)	Delay (s)	Unicast End to End Throughput (bits/s)	Unicast End to End Delay (s)	Throughput (bits/s)	Delay (s)	Jitter (s)	Hop count	Queue Length (in bytes)	Longest Time in Queue
**IEEE 9 (CBR node 7 to node 6)**											
**Proposed agent-based approach**		3400	0.00131	4400	0.0013	1200	0.0011	5.5 × 10^−8^	5	9 × 10^−6^	5 × 10^−6^
**No agent involvement**	**RIP protocol**	4400	0.0073	3200	0.0073	800	0.0013	0.00017	3	0.0009	0.00044
	**OLSR**	3050	0.0073	4400	0.0073	2000	0.0012	5 × 10^6^	1	0.0009	0.00045
	**OSPFv2**	2700	0.0074	4600	0.0074	1700	0.0013	5.5 × 10^6^	5	0.0009	0.00026
**IEEE 14 (CBR node 2 to node 14)**											
**Proposed agent-based approach**		3450	0.0073	4250	0.0073	9000	0.0013	0.000019	2	0.008	0.00043
**No agent involvement**	**RIP protocol**	3400	0.0074	4200	0.0074	9000	0.00134	0.000185	2	0.0085	0.00044
	**OLSR**	3050	0.0074	4400	0.0074	9700	0.0015	7.5 × 10^6^	1	0.01	0.0006
	**OSPFv2**	2700	0.0074	4500	0.0074	3700	0.0017	0.00055	3	0.034	0.0095
**IEEE 34 (CBR node 17 to node 26)**											
**Proposed agent-based approach**		3450	0.0145	4300	0.014	11,600	0.001	0.000185	3	0.0095	0.00055
**No agent involvement**	**RIP protocol**	2100	0.015	5100	0.00145	3500	0.0014	0.0002	2	0.0135	0.00065
	**OLSR**	2900	0.0145 Jitter (1.7 × 10^−5^)	4400	0.0145 Jitter (1.7 × 10^−5^)	13,000	0.0011	0.00055	1	0.0115	0.00065
	**OSPFv2**	1850	0.0155	5500	0.0145	3,200	0.0011	0.00055	4	0.08	0.0097
**IEEE 39 (CBR node 18 to node 15)**											
**Proposed agent-based approach**		3450	0.0013	4400	0.0013	9000	0.000125	0.00015	3	0.01	0.000457
**No agent involvement**	**RIP protocol**	2900	0.0073	4450	0.0073	2200	0.0014	0.0002	3	0.013	0.00075
	**OLSR**	3000	0.0073	4450	0.0073	46,000	0.0011	0.0006	1	0.024	0.00061
	**OSPFv2**	1850	0.0073	5500	0.0073	4500	0.001	0.0006	2	1.1	0.019
**IEEE 57 (CBR node 38 to node 46)**											
**Proposed agent-based approach**		3450	0.00145	4450	0.0014	19,000	0.00014	0.00015	3	0.009	0.00059
**No agent involvement**	**RIP protocol**	2200	0.0023	5100	0.00145	3500	0.0014	0.0002	2	0.0135	0.00065
	**OLSR**	2950	0.0155 Jitter (1.7 × 10^−6^)	4600	0.0155 Jitter (1.7 × 10^−6^)	21,000	0.0012	0.00045	1	0.0135	0.00075
	**OSPFv2**	1250	0.0135	5500	0.0145	13,200	0.0012	0.00045	4	0.089	0.0095

**Table 2:** Comparative analysis of performance shown in Table 1.

Microgrid Bus System (100 Mbps bandwidth)		UDP: Unicast		CBR Server		UDP: Broadcast					
		Throughput (bits/s)	Delay (s)	Unicast End to End Throughput (bits/s)	Unicast End to End Delay (s)	Throughput (bits/s)	Delay (s)	Jitter (s)	Hop Count	Queue Length (in bytes)	Longest Time in Queue
**IEEE 9**											
**Proposed agent-based approach**			✓		✓		✓	✓		✓	✓
**No agent involvement**	**RIP protocol**										
	**OLSR**					✓			✓		
	**OSPFv2**	✓		✓							
**IEEE 14**											
**Proposed agent-based approach**		✓	✓		✓		✓	✓		✓	✓
**No agent involvement**	**RIP protocol**										
	**OLSR**					✓			✓		
	**OSPFv2**			✓							
**IEEE 34**											
**Proposed agent-based approach**		✓	✓		✓		✓	✓		✓	✓
**No agent involvement**	**RIP protocol**										
	**OLSR**					✓			✓		
	**OSPFv2**			✓							
**IEEE 39**											
**Proposed agent-based approach**		✓	✓		✓		✓	✓		✓	✓
**No agent involvement**	**RIP protocol**										
	**OLSR**					✓			✓		
	**OSPFv2**			✓							
**IEEE 57**											
**Proposed agent-based approach**		✓	✓		✓		✓	✓		✓	✓
**No agent involvement**	**RIP protocol**										
	**OLSR**					✓			✓		
	**OSPFv2**			✓							

**Table 3:** Performance evaluation of the bus system for fault case study 1 in the microgrid communication network.

Microgrid Bus System (100 Mbps bandwidth)		UDP: Unicast		CBR Server		UDP: Broadcast					
		Throughput (bits/s)	Delay (s)	Unicast End to End Throughput (bits/s)	Unicast End to End Delay (s)	Throughput (bits/s)	Delay (s)	Jitter (s)	Hop Count	Queue Length (in bytes)	Longest Time in Queue
**IEEE 9 (fault injected in node 7)**											
**Proposed agent-based approach**		3500	0.00131	4300	0.0013	1200	0.0011	5.5 × 10^−8^	4	9 × 10^−6^	5 × 10^−6^
**No agent involvement**	**RIP protocol**	4400	0.0073	3200	0.0073	800	0.0012	0.00017	2	0.0009	0.00045
	**OLSR**	3050	0.0073	4400	0.0073	2000	0.0014	5 × 10^−6^	2	0.0009	0.00045
	**OSPFv2**	2700	0.0074	4700	0.0074	1700	0.0013	5.5 × 10^−6^	5	0.0009	0.00026
**IEEE 14 (fault injected in node 2)**											
**Proposed agent-based approach**		3450	0.0073	4250	0.0072	9000	0.0013	0.000019	2	0.008	0.00043
**No agent involvement**	**RIP protocol**	3350	0.0074	4200	0.0074	8500	0.00114	0.000185	1	0.0085	0.00044
	**OLSR**	3050	0.0075	4400	0.0074	9000	0.0015	7.5 × 10^−6^	1	0.01	0.0006
	**OSPFv2**	2700	0.0074	4500	0.0074	3700	0.0017	0.00055	3	0.035	0.0095
**IEEE 34 (fault injected in node 17)**											
**Proposed agent-based approach**		3450	0.015	4300	0.014	11,600	0.001	0.000185	2	0.0095	0.00055
**No agent involvement**	**RIP protocol**	2100	0.015	5100	0.00155	3500	0.0014	0.0002	2	0.0135	0.00065
	**OLSR**	2800	0.0145 Jitter (1.7 × 10^−5^)	4400	0.0145 Jitter (1.7 × 10^−5^)	13,000	0.0011	0.00055	1	0.0115	0.00065
	**OSPFv2**	1850	0.0155	5500	0.0145	3200	0.0011	0.00055	4	0.08	0.0097
**IEEE 39 (fault injected in node 18)**											
**Proposed agent-based approach**		3450	0.0013	4400	0.0013	9000	0.000125	0.00015	3	0.01	0.000457
**No agent involvement**	**RIP protocol**	2900	0.0073	4450	0.00735	2200	0.0014	0.0002	4	0.013	0.00075
	**OLSR**	3100	0.0073	4450	0.0073	46,000	0.0011	0.0006	1	0.024	0.00061
	**OSPFv2**	1850	0.0073	5500	0.0073	4500	0.001	0.0006	2	1.1	0.019
**IEEE 57 (fault injected in node 38)**											
**Proposed agent-based approach**		3450	0.00145	4450	0.0014	19,000	0.00014	0.00015	3	0.009	0.00059
**No agent involvement**	**RIP protocol**	3450	0.00145	4450	0.0014	19,000	0.00011.5	0.00015	3	0.009	0.00059
	**OLSR**	2300	0.0023	5100	0.00145	3500	0.0014	0.0002	2	0.0135	0.00065
	**OSPFv2**	2950	0.0155 Jitter (1.7 × 10^−6^)	4600	0.0155 Jitter (1.7 × 10^−6^)	21,000	0.0012	0.00045	1	0.0145	0.00075

* On comparing the changes with Table 1, the visible changes are highlighted using red color.

**Table 4:** Performance evaluation of bus system for the fault case study 2 in the microgrid communication network.

Microgrid Bus System (100 Mbps bandwidth)		UDP: Unicast		CBR Server		UDP: Broadcast					
		Throughput (bits/s)	Delay (s)	Unicast End to End Throughput (bits/s)	Unicast End to End Delay (s)	Throughput (bits/s)	Delay (s)	Jitter (s)	Hop Count	Queue Length (in bytes)	Longest Time in Queue
**IEEE 9 (fault injected in node 7 and 8)**											
**Proposed agent-based approach**		3500	0.00131	4300	0.0013	1200	0.0011	5.5 × 10^−8^	4	9 × 10^−6^	5 × 10^−6^
**No agent involvement**	**RIP protocol**	4200	0.0074	3200	0.0073	800	0.0013	0.00017	4	0.0009	0.00045
	**OLSR**	3050	0.0073	4400	0.0073	2300	0.0014	5 × 10^−6^	2	0.0009	0.00047
	**OSPFv2**	2500	0.0074	4700	0.0074	1700	0.0013	5.5 × 10^−6^	5	0.0009	0.00026
**IEEE 14 (fault injected in node 2 and 5)**											
**Proposed agent-based approach**		3450	0.0073	4250	0.0072	9000	0.0013	0.000019	2	0.008	0.00043
**No agent involvement**	**RIP protocol**	3350	0.0074	4100	0.0074	8500	0.00114	0.000185	1	0.0085	0.00044
	**OLSR**	3050	0.0075	4400	0.0074	9000	0.0015	7.5 × 10^−6^	1	0.01	0.00062
	**OSPFv2**	2700	0.0074	4500	0.0075	3700	0.0017	0.00055	3	0.035	0.0095
**IEEE 34 (fault injected in node 17 and 6)**											
**Proposed agent-based approach**		3450	0.015	4300	0.014	11,600	0.001	0.000185	2	0.0095	0.00055
**No agent involvement**	**RIP protocol**	2100	0.015	5100	0.00155	3500	0.0014	0.0002	3	0.0135	0.0007
	**OLSR**	2800	0.0145 Jitter (1.7 × 10^−5^)	4400	0.0145 Jitter (1.7 × 10^−5^)	14,000	0.0012	0.00055	1	0.0115	0.00065
	**OSPFv2**	1850	0.0155	5500	0.0145	3200	0.0011	0.00055	5	0.08	0.0097
**IEEE 39 (fault injected in node 18 and 17)**											
**Proposed agent-based approach**		3450	0.0013	4400	0.0013	9000	0.000125	0.00015	3	0.01	0.000457
**No agent involvement**	**RIP protocol**	2900	0.0075	4500	0.00735	2200	0.0014	0.0002	4	0.013	0.00075
	**OLSR**	3100	0.0073	4450	0.0073	45,000	0.0011	0.0006	1	0.024	0.00061
	**OSPFv2**	1850	0.0073	5500	0.0073	4500	0.001	0.0006	2	1.1	0.0192
**IEEE 57 (fault injected in node 38 and 27)**											
**Proposed agent-based approach**		3550	0.00145	4450	0.00135	19,000	0.00014	0.00015	3	0.009	0.00059
**No agent involvement**	**RIP protocol**	3450	0.00145	4450	0.0014	19,000	0.00013	0.00015	3	0.009	0.00059
	**OLSR**	2300	0.0023	5100	0.00145	3500	0.0014	0.0002	2	0.0135	0.00065
	**OSPFv2**	2950	0.0155 Jitter ( 1.8 × 10^−6^ )	4600	0.0155 Jitter (1.7 × 10^−6^)	21,000	0.0012	0.00055	1	0.0145	0.00075

* The table highlights the changes compared with Table 3 and the visible changes are highlighted using violet color in this table.
